# Impact of Perioperative Blood Transfusion on Postoperative Complications and Prognosis of Gastric Adenocarcinoma Patients with Different Preoperative Hemoglobin Value

**DOI:** 10.1155/2016/6470857

**Published:** 2015-12-27

**Authors:** Lian Xue, Xiao-Long Chen, Zhang Wei-Han, Kun Yang, Xin-Zu Chen, Bo Zhang, Zhi-Xin Chen, Jia-Ping Chen, Zong-Guang Zhou, Jian-Kun Hu

**Affiliations:** ^1^Department of Gastrointestinal Surgery, West China Hospital, Sichuan University, No. 37 Guo Xue Xiang Street, Chengdu, Sichuan 610041, China; ^2^Laboratory of Gastric Cancer, State Key Laboratory of Biotherapy, West China Hospital, Sichuan University, No. 37 Guo Xue Xiang Street, Chengdu, Sichuan 610041, China; ^3^Laboratory of Digestive Surgery, State Key Laboratory of Biotherapy, West China Hospital, Sichuan University, No. 37 Guo Xue Xiang Street, Chengdu, Sichuan 610041, China

## Abstract

*Background*. The impact of perioperative blood transfusion on the prognosis of gastric adenocarcinoma patients is still controversial. The aim of this study was to elucidate the impact of perioperative blood transfusion on postoperative complications and prognosis of patients who underwent gastrectomy for gastric adenocarcinoma with different levels of preoperative hemoglobin value (POHb). *Method*. From 2003 to 2011, 1199 patients who underwent curative gastrectomy were retrospectively enrolled and followed up to December 2014. Clinicopathological features and survival outcomes were compared between transfused and nontransfused patients. *Results*. In this study, transfused patients had more postoperative complications than nontransfused ones (*P* = 0.002). In survival analysis, the difference was not significant between transfused and nontransfused patients with POHb between 70 and 100 g/L (*P* = 0.191). However, in patients with POHb >100 g/L, transfused patients had significantly worse prognosis (*P* < 0.001), especially in TNM III stage patients (*P* = 0.002). And intraoperative blood transfusion predicted poor prognosis (*P* = 0.001). *Conclusion*. Perioperative blood transfusion might lead to poor survival in gastric adenocarcinoma patients with POHb >100 g/L and transfused patients had more postoperative complications; thus it is better to refrain from unnecessary perioperative blood transfusion especially intraoperative transfusion.

## 1. Introduction

Gastric adenocarcinoma (GAC) is one of the most common malignant cancers in the world [1]. Surgery is considered to be the main treatment for GAC. Patients with cancers were prone to have anemia, and radical resection with extended lymphadenectomy might cause much blood loss during surgery; thus patients with low hemoglobin (Hb) value might receive perioperative blood transfusion.

Whether perioperative blood transfusion impacted on the prognosis of patients with cancer was still under debate. Some studies reported that blood transfusion was related to poor prognosis of patients with colorectal cancer, renal cell carcinoma, lung cancer, and breast cancer [2–6]. The possible reason might be that blood transfusion could cause immunomodulation, which decreased the activities of natural killer cells and increased the activities of regulatory T cells [7]. However, other studies held the opposite opinion, indicating that blood transfusion did not affect patient's outcomes [8, 9].

With respect to gastric adenocarcinoma, several researches had been carried out in order to find out the relationship among perioperative blood transfusion, postoperative complications, and prognosis, but it still remained controversial. Although some reports did not support the fact that blood transfusion was related to poor prognosis, others claimed that it did exist [10–14]. Some reports showed that transfused patients had more postoperative complications [13, 15].

In China, according to the clinical guideline of blood transfusion, patients with POHb lower than 70 g/L should be transfused, and there is no need of transfusion for patients with POHb >100 g/L [16]. However, there were few studies discussing prognosis of perioperative transfused patients with cancer grouped by different POHb. In our study, we divided the patients into different groups according to their POHb and tried to find out the relationship among blood transfusion, postoperative complications, and survival of patients in these different groups.

## 2. Methods

The West China Hospital research ethics committee approved retrospective analysis of anonymous data. Signed patient informed consent was waived per the committee approval, because it was a retrospective analysis.

### 2.1. Patients

From January 2003 to April 2011, 1199 patients who underwent curative gastrectomy with R0 resection for gastric adenocarcinoma at Department of Gastrointestinal Surgery, West China Hospital, Sichuan University, were enrolled in our study. Patients with distant metastases, neoadjuvant chemoradiotherapy, previous neoplastic diseases, and hematological pathologies were excluded from our study. We divided patients into three groups according to their POHb value: Group 1: patients with POHb ≤70 g/L; Group 2: patients with POHb between 70 and 100 g/L; Group 3: patients with POHb >100 g/L. In Group 2 and Group 3, patients were divided into two subgroups according to whether they had perioperative blood transfusion. And all the patients in Group 1 had received blood transfusion. The clinicopathological features such as tumor size, tumor location, differentiation grade, and pathological TNM stage were recorded according to Japanese classification of gastric carcinoma by JGCA [17]. Other variables including operation time, intraoperative blood loss, and postoperative complications were also analyzed. Clinicopathological features and overall survival rates were compared among these groups.

### 2.2. Surgical Treatment

Gastrectomy plus lymphadenectomy was the mainstay treatment for patients with gastric adenocarcinoma. Patients with early gastric adenocarcinoma underwent D1/D1+, while patients with advanced gastric adenocarcinoma underwent D2/D2+ lymphadenectomy. All the operations were performed by expertise of surgeons specialized in gastrointestinal surgery according to Japanese Gastric Cancer Treatment Guidelines [18].

### 2.3. Perioperative Blood Transfusions

Perioperative blood transfusion was defined as either whole blood or packed red blood cells administered within 14 days before surgery, during surgery, or 14 days after surgery [14]. Usually, POHb ≤ 70 g/L and significant intraoperative blood loss were general indications for blood transfusion. The amount and time of perioperative blood transfusion were recorded.

### 2.4. Follow-Up

Regular outpatient visit was the first choice for follow-up, while telephones and mails were adopted as two main supplementary follow-up methods. Follow-up information was updated until December 2014. The main reasons for the loss of follow-up were the changes of phone number or home address and refusal of reexamination in our hospital.

### 2.5. Statistical Analysis

All the statistical analyses were performed with the statistical software SPSS 19.0 (SPSS, Chicago, Illinois, USA). All continuous values were presented as mean ± standard deviation (SD). Unordered categorical variable and ranked data were analyzed through chi-square test and rank sum test, respectively. Student's *t*-test was used to analyze continuous data if variance was homogeneity and distribution was normal. If not, rank sum test was used. Multivariate logistic regression analysis was performed to identify risk factors for need of perioperative blood transfusion. Survival curves were computed using Kaplan-Meier and compared by means of log-rank test. Multivariate analysis was performed by Cox regression test to find out independent prognostic factors. *P* value < 0.05 was considered statistically significant.

## 3. Results

### 3.1. Demography of Patients

In this study, 1199 patients with different POHb were included, with 345 (28.8%) patients receiving perioperative blood transfusion and 854 (71.2%) receiving no transfusion. 54 (4.5%) patients were in POHb ≤ 70 g/L group while 257 (21.4%) ones were in POHb between 70 and 100 g/L group and 888 (74.1%) patients in POHb >100 g/L group. Among transfused patients, 177 (51.3%) patients received less than 2 units (200 mL/unit) of blood transfusion, 94 (27.2%) patients received 2–4 units, and 74 (21.5%) received more than 4 units. Ninety-six (27.8%) patients underwent preoperative transfusion; 210 (60.9%) ones underwent intraoperative transfusion while 39 (11.3%) patients underwent postoperative transfusion.

Comparisons of clinicopathological features between transfused and nontransfused patients were shown in Table 1. Of all the patients, transfused ones had significantly more combined organ resections (*P* < 0.001), more patients with open surgery (*P* < 0.001), and more intraoperative blood loss (*P* < 0.001). Postoperative complications seemed to occur more frequently in transfused patients (*P* = 0.002) especially pulmonary infection (transfused 18.8% versus nontransfused 13.7%).  Table 2 showed the comparison between transfused and nontransfused patients in POHb between 70 and 100 g/L or POHb >100 g/L groups. Clinicopathological characteristics were similar with regard to sex, TNM stage, differentiation grade, macroscopic type, and tumor size in transfused and nontransfused subgroups of patients with POHb between 70 and 100 g/L (all *P* > 0.05). However, in the patients with POHb >100 g/L group, transfused patients had more advanced TNM stage (*P* < 0.001), larger tumor size (*P* = 0.009), more poor differentiated grade (*P* = 0.010), and more postoperative complications (*P* = 0.027).

### 3.2. Risk Factors for Blood Transfusion

To identify risk factors for blood transfusion, univariate and multivariate analyses were performed in each group, respectively. The results of multivariate analysis of different groups were shown in Tables 3 and 4. In logistic regression analysis, risk factors for blood transfusion in patients with POHb between 70 and 100 g/L were significantly associated with intraoperative blood loss (*P* = 0.002) and combined organ resection (*P* = 0.015). However, risk factors for blood transfusion in patients with POHb >100 g/L were associated with age (*P* = 0.013), combined organ resection (*P* = 0.033), TNM stage (*P* = 0.005), and intraoperative blood loss (*P* < 0.001).

### 3.3. Survival Analyses

Finally, 999 patients (83.3%) were followed up and included in survival analysis. The median survival time of transfused and nontransfused group was 46.3 (0.07–141.9) months and 111.5 (0–141.9) months, respectively. Three-year survival rates were 51% and 61% in transfused and nontransfused patients with POHb between 70 and 100 g/L, respectively. In transfused and nontransfused patients with POHb >100 g/L, the 3-year survival rates were 44% and 65%, respectively. In univariate analysis, age (*P* = 0.001), type of gastrectomy (*P* < 0.001), combined organ resections (*P* = 0.003), longitudinal location (*P* < 0.001), differentiation grade (*P* = 0.009), macroscopic type (*P* < 0.001), tumor size (*P* < 0.001), pT stage (*P* < 0.001), pN stage (*P* < 0.001), pTNM stage (*P* < 0.001), and perioperative blood transfusion (*P* < 0.001) were associated with overall survival in all patients with gastrectomy in our study (Table 5). The multivariate analysis revealed that age (*P* = 0.005), tumor size (*P* < 0.001), and TNM stage (*P* < 0.001) were independent prognostic factors in gastric adenocarcinoma patients (Table 5).

### 3.4. Subgroup Analyses

**Figure 1:**
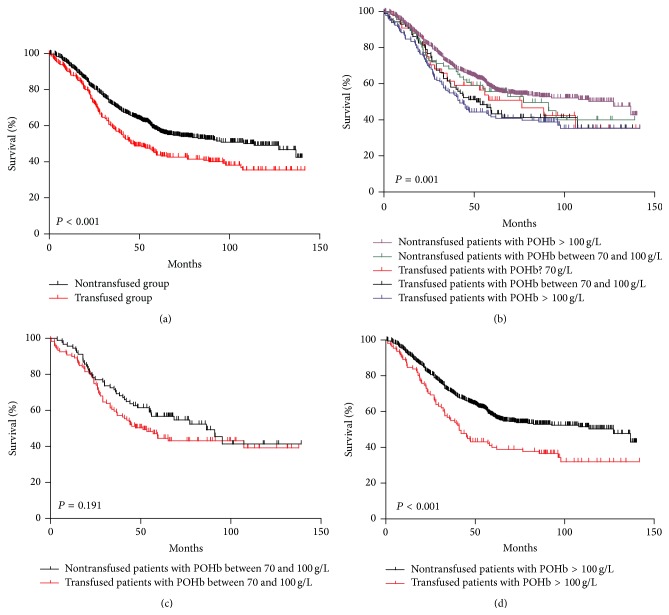
(a) Survival analysis of transfused and nontransfused patients. (b) Survival analysis of transfused and nontransfused patients with different POHb. (c) Survival analysis of transfused and nontransfused patients with POHb between 70 and 100 g/L. (d) Survival analysis of transfused and nontransfused patients with POHb >100 g/L.

**Figure 2:**
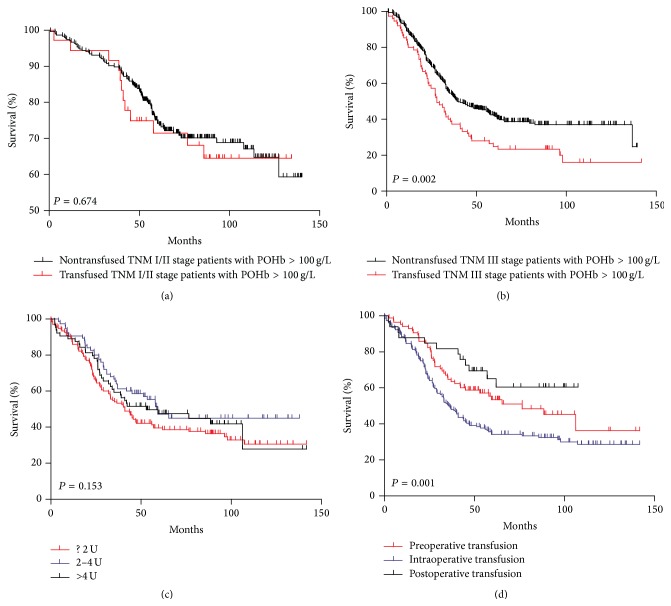
(a) Survival analysis of transfused and nontransfused TNM I/II stage patients with POHb >100 g/L. (b) Survival analysis of transfused and nontransfused TNM III stage patients with POHb >100 g/L. (c) Survival analysis of transfused patients with different units of blood. (d) Survival analysis of transfused patients with different transfusion time.

Of all patients, Kaplan-Meier curve showed that transfused patients had significantly worse prognosis than nontransfused patients (Figure 1(a), *P* < 0.001). Nontransfused patients with POHb >100 g/L had better survival outcomes among the subgroups we divided in this study (Figure 1(b), *P* = 0.001). When comparing prognosis between transfused and nontransfused patients in POHb between 70 and 100 g/L and POHb >100 g/L groups, respectively, we found out that there was no significant difference in prognosis between transfused and nontransfused ones in POHb between 70 and 100 g/L group (*P* = 0.191, Figure 1(c)). However, in POHb >100 g/L group, the survival was significantly better in the nontransfused group than transfused group (*P* < 0.001, Figure 1(d)). We further divided patients according to their TNM stage (I/II stage; III stage) in POHb >100 g/L subgroup, and constituent ratios of differentiation grade, macroscopic type, and tumor size between these two subgroups were similar. We found that, in stage I/II subgroup, the difference was not significant between transfused and nontransfused groups (*P* = 0.674, Figure 2(a)); however, in TNM stage III subgroup, survival outcome was remarkably better in nontransfused group than that in transfused group (*P* = 0.002, Figure 2(b)). In addition, a dose-response relationship between the amount of transfused blood and prognosis was not recognized (*P* = 0.153, Figure 2(c)), and the patients with intraoperative transfusion had obviously worse prognosis than those who had preoperative or postoperative transfusion (*P* = 0.001, Figure 2(d)).

## 4. Discussion

Variety of experiments had demonstrated that perioperative blood transfusion could result in immunological changes which might contribute to poor survival of patients [19, 20]. Although many studies had been carried out to evaluate the influence of perioperative blood transfusion on the prognosis of gastric adenocarcinoma patients, the results were still varied. Sánchez-Bueno et al.'s study showed that perioperative blood transfusion did not influence the survival of patients with gastric adenocarcinoma [11]. A study by Moriguchi et al. also revealed the lack of any relationship between perioperative blood transfusion and survival of patients with gastric cancer [21]. On the other hand, other studies had opposite opinion that blood transfusion resulted in worse prognosis of gastric cancer patients. Ojima et al. demonstrated that perioperative blood transfusion had a negative influence on survival of patients with gastric cancer [22]. A large retrospective study with 1710 patients by Hyung et al. found that blood transfusion was an independent risk factor for recurrence and poor survival [14]. However, all the studies mentioned above discussed the relationship between perioperative blood transfusion and prognosis without concerning POHb of patients. Clinical decision for blood transfusion was driven more by hemoglobin threshold. Whether patients with POHb >70 g/L should be transfused and whether perioperative transfusion could influence postoperative complications and prognosis of gastric adenocarcinoma patients with different POHb still remain unknown. Therefore, in our study, we divided gastric adenocarcinoma patients into different groups according to their POHb and tried to find out the impact of transfusion on postoperative complications and prognosis.

Our retrospective study showed that transfused patients had more postoperative complications than nontransfused ones. Other studies also demonstrated that transfused gastric cancer patients had more postoperative complications [13, 15]. We also found that pulmonary infection happened more frequently in transfused patients than nontransfused ones (transfused 18.8% versus nontransfused 13.7%). Maybe transfusion could disturb the immune system and cause high morbidity of pulmonary infection.

In our study, perioperative blood transfusion resulted in poor prognosis of patients with gastric adenocarcinoma but not an independent prognostic factor. However, this might be influenced by different distributions of clinicopathological features or operation approach between transfused and nontransfused subgroups. When patients were divided according to POHb, we found out that difference was not significant between prognosis of transfused and nontransfused patients with POHb between 70 and 100 g/L (*P* = 0.191). Survival outcome was significantly worse in transfused group than nontransfused group in patients with POHb >100 g/L; however, this difference might be caused by more advanced TNM stage patients in transfused group. Hence, we carried out survival analysis grouped by TNM stage (I/II stage; III stage) in patients with POHb >100 g/L. Distributions were similar with regard to sex, differentiation grade, macroscopic type, and tumor size in both subgroups. We found that transfused TNM III stage patients had worse prognosis than nontransfused patients, while differences were not significant between transfused and nontransfused patients in I/II stage subgroups. We concluded that perioperative blood transfusion contributed to poor prognosis, especially in stage III gastric adenocarcinoma patients.

Blood transfusion may regulate immunity system which may be harmful for patients with cancer. The explanation for the adverse effect of transfusion is probably immunosuppression which may be caused by decreased natural killer cell activity and increased suppressor T lymphocytes activity. Other suppressor factors such as anti-idiotypic antibodies may also be generated after blood transfusion [7, 23]. Heiss held the opinion that transfusion might have effect on minimal residual disease in resected-cancer patients which might lead to poor prognosis of them [24]. They witnessed a significant quantitative increase of tumor cells in bone marrow of transfused patients only during follow-up. In advanced stage, immunosuppression caused by transfusion may lead to progression of residual foci and also fail to clear cancer cells in bone marrow. Their finding supported our results that transfused patients had worse prognosis and more postoperative complications such as pulmonary infections, especially in TNM stage III patients.

There were some reports focused on the relationship between prognosis and amount of transfused blood. Hyung's study described significant differences in the prognosis between patients transfused with different units of blood [14]. However, in this study, we revealed that transfused patients with different units of blood did not show significant difference in survival outcomes which was similar to Ojima's and Kanda's results [15, 22]. Maybe it was transfusion itself which caused immunosuppression and poor prognosis rather than amount of blood transfused. We also found that patients with intraoperative transfusion seemed to have worse survival than preoperative and postoperative transfused patients. Surgical stress inhibited the immune system and might result in immunosuppression which led to poor prognosis of patients with intraoperative blood transfusion [25]. If we cannot avoid perioperative transfusion, we may avoid intraoperative transfusion at least.

This present study, which is one of the largest retrospective researches on Chinese patients, aims to excavate the relationship among Hb value, transfusion, and prognosis. In patients with POHb between 70 and 100 g/L, the difference was not significant between transfused and nontransfused patients. However, in patients with POHb >100 g/L especially TNM III stage, perioperative blood transfusion related to poor outcomes. Although transfusion did not affect much prognosis of patients with POHb between 70 and 100 g/L, blood transfusion still should be avoided for transfused patients who had more postoperative complications. And we also should avoid intraoperative blood transfusion for the adverse effect on prognosis. In our study, intraoperative blood loss was an independent risk factor for transfusion. Thus, as to surgeons, the best method to avoid perioperative transfusion is to reduce intraoperative blood loss, for example, careful anatomical dissection to avoid injury of blood vessels; using some technology or devices to reduce blood loss such as electrocoagulation, ultrasonic, laser devices, and collagen-sealing devices; reducing the length of incision and suture carefully.

Our study is a retrospective study which included 1199 Chinese patients in one center, and we did not analyze the effect of postoperative treatment such as adjuvant chemotherapy or radiotherapy on the long-term survival of our patients in this study. Thus it is necessary to carry out some prospective, randomized, controlled studies to examine the prognostic value of blood transfusion in gastric adenocarcinoma.

In conclusion, perioperative blood transfusion was related to poor prognosis of patients with gastric adenocarcinoma, especially in TNM III stage patients with POHb >100 g/L. Transfused patients also had more postoperative complications than nontransfused ones; thus it is better to refrain from unnecessary perioperative blood transfusion especially intraoperative transfusion.

## Figures and Tables

**Table 1:** Comparison of clinicopathological features of all transfused and nontransfused patients.

	Categories	Transfused	Nontransfused	*P* value
		*n* = 345 (%)	*n* = 854 (%)	
Age	Mean ± SD	58.4 ± 11.7	55.9 ± 11.5	0.001

Gender	Male	244 (70.7)	605 (70.8)	0.967
	Female	101 (29.3)	245 (29.2)	

Combined organ resection	Yes	41 (11.9)	31 (3.6)	<0.001
	No	304 (88.1)	823 (96.4)	

Type of gastrectomy	DG	193 (55.9)	519 (60.8)	0.055
	TG	86 (24.9)	160 (18.7)	
	PG	66 (19.2)	175 (20.5)	

Operation approach	Open surgery	325 (94.2)	725 (84.9)	<0.001
	Laparoscopic assisted	20 (5.8)	129 (15.1)	

Concomitant diseases	Yes	182 (52.8)	403 (47.2)	0.081
	No	163 (47.2)	451 (52.8)	

Longitudinal location	U	72 (20.9)	221 (25.9)	0.004
	M	63 (18.3)	102 (11.9)	
	L	202 (58.6)	523 (61.2)	
	Whole	8 (2.2)	8 (1.0)	

Differentiation grade	Well/moderate	48 (13.9)	171 (20.0)	0.013
	Poor/undifferentiated	297 (86.1)	683 (80.0)	

Macroscopic type	Types 0–2	210 (60.9)	569 (66.6)	0.058
	Types 3–4	135 (39.1)	285 (33.4)	

Tumor size (cm)	Mean ± SD	5.7 ± 2.6	4.6 ± 2.5	<0.001
	≤4.5	120 (34.8)	461 (54.0)	<0.001
	>4.5	225 (65.2)	393 (46.0)	

T stage	T1	28 (8.1)	192 (22.5)	<0.001
	T2	37 (10.7)	121 (14.2)	
	T3	14 (4.1)	44 (5.2)	
	T4	266 (77.1)	497 (58.1)	

N stage	N0	84 (24.3)	319 (37.4)	<0.001
	N1	69 (20.0)	154 (18.0)	
	N2	66 (19.1)	168 (19.7)	
	N3a	90 (26.1)	157 (18.4)	
	N3b	37 (10.5)	56 (6.5)	

TNM stage	I/II	108 (31.3)	410 (48.0)	<0.001
	III	237 (68.7)	444 (52.0)	

Operation time (min)	Mean ± SD	241.0 ± 61.9	232.8 ± 49.9	0.169

Intraoperative blood loss (mL)	Mean ± SD	528.7 ± 282.2	414.2 ± 194.3	<0.001

Postoperative complications	Stomach without tension	2 (0.6)	19 (2.2)	0.002
	Wound infection	7 (2.0)	10 (1.2)	
	Anastomotic leakage	8 (2.3)	6 (0.7)	
	Bleeding	9 (2.6)	3 (0.4)	
	Pulmonary infection	65 (18.8)	117 (13.7)	
	Others	14 (4.1)	16 (1.9)	
	Total patients	82 (23.8)	152 (17.8)	

Postoperative hospital stay	Mean ± SD	12.4 ± 10.6	11.7 ± 9.8	0.261

SD: standard deviation, DG: distal gastrectomy, TG: total gastrectomy, PG: proximal gastrectomy, U: upper, M: middle, and L: lower.

**Table 2:** Comparison of clinicopathological features of transfused and nontransfused patients in POHb between 70 and 100 g/L or >100 g/L subgroups.

	Categories	Transfused ones with POHb between 70 and 100 g/L	Nontransfused with POHb between 70 and 100 g/L	*P* value	Transfused ones with POHb >100 g/L	Nontransfused with POHb >100 g/L	*P* value
		*n* = 150 (%)	*n* = 107 (%)		*n* = 141 (%)	*n* = 747 (%)	
Age	Mean ± SD	60.2 ± 10.8	57.4 ± 12.6	0.051	58.1 ± 11.2	55.7 ± 11.4	0.020

Gender	Male	108 (72.0)	69 (64.5)	0.200	100 (70.9)	536 (71.8)	0.841
	Female	42 (28.0)	38 (35.5)		41 (29.1)	211 (28.2)	

Combined organ resection	Yes	16 (10.7)	1 (0.9)	0.002	22 (15.6)	30 (4.0)	<0.001
	No	134 (89.3)	106 (99.1)		119 (84.4)	717 (96.0)	

Type of gastrectomy	DG	83 (55.3)	62 (57.9)	0.916	71 (50.4)	457 (61.2)	0.024
	TG	39 (26)	26 (24.3)		38 (27.0)	134 (17.9)	
	PG	28 (18.7)	19 (17.8)		32 (22.6)	156 (20.9)	

Open approach	Open surgery	141 (94.0)	93 (86.9)	0.050	138 (97.9)	632 (84.6)	<0.001
	Laparoscopic assisted	9 (6.0)	14 (13.1)		3 (2.1)	115 (15.4)	

Longitudinal location	U	30 (20.0)	27 (25.2)	0.279	34 (24.1)	194 (26.0)	0.024
	M	26 (17.3)	16 (15.0)		27 (19.1)	86 (11.5)	
	L	90 (60.0)	64 (59.8)		76 (53.9)	459 (61.4)	
	Whole	4 (2.7)	0 (0)		4 (2.9)	8 (1.1)	

Differentiation grade	Well/moderate	22 (14.7)	17 (15.9)	0.788	16 (11.3)	154 (20.6)	0.010
	Poor/undifferentiated	128 (85.7)	90 (84.1)		125 (88.7)	593 (79.4)	

Macroscopic type	Types 0–2	91 (60.7)	64 (59.8)	0.890	86 (61.0)	505 (67.6)	0.127
	Types 3–4	59 (39.3)	43 (40.2)		55 (39.0)	242 (32.4)	

Tumor size (cm)	Mean ± SD	5.9 ± 2.6	5.5 ± 3.0	0.262	5.1 ± 2.5	4.5 ± 2.3	0.009
	≤4.5	47 (31.3)	41 (38.3)	0.245	60 (42.6)	420 (56.2)	
	>4.5	103 (68.7)	66 (61.7)		81 (57.4)	327 (43.8)	

T stage	T1	12 (8.0)	15 (14.0)	0.227	15 (10.6)	177 (23.7)	<0.001
	T2	20 (13.3)	16 (15.0)		12 (8.5)	105 (14.1)	
	T3	6 (4.0)	2 (1.9)		5 (3.5)	42 (5.6)	
	T4	112 (74.7)	74 (69.1)		109 (77.4)	423 (56.6)	

N stage	N0	35 (23.4)	32 (29.9)	0.228	34 (24.1)	287 (38.4)	<0.001
	N1	32 (21.3)	20 (18.7)		26 (18.4)	134 (18.0)	
	N2	32 (21.3)	26 (24.3)		27 (19.1)	142 (19.0)	
	N3a	36 (24.0)	21 (19.6)		38 (27.0)	136 (18.2)	
	N3b	15 (10.0)	8 (7.5)		16 (11.4)	48 (6.4)	

TNM stage	I/II	49 (32.7)	46 (43.0)	0.091	44 (31.2)	364 (48.7)	<0.001
	III	101 (67.3)	61 (57.0)		97 (68.8)	383 (51.3)	

Operation time (min)	Mean ± SD	236.9 ± 55.2	228.4 ± 51.4	0.231	249.3 ± 64.6	233.5 ± 49.6	0.008

Intraoperative blood loss (mL)	Mean ± SD	498.5 ± 215.2	408.5 ± 212.1	0.001	625.9 ± 421.5	415.0 ± 191.7	0.002

Postoperative complications	Yes	32 (21.3)	18 (16.8)	0.426	37 (26.2)	134 (17.9)	0.027
	No	118 (78.7)	89 (83.2)		104 (73.8)	613 (82.1)	

Postoperative hospital stay	Mean ± SD	12.0 ± 8.4	11.2 ± 6.7	0.428	13.2 ± 13.7	11.7 ± 10.2	0.146

SD: standard deviation, DG: distal gastrectomy, TG: total gastrectomy, PG: proximal gastrectomy, U: upper, M: middle, and L: lower.

**Table 3:** Multivariate analysis of risk factors for blood transfusion in patients with POHb between 70 and 100 g/L.

	Parameter estimate	SE	Adjusted OR	95% CI	*P* value
Age					0.073
Gender					0.097
Macroscopic type					0.256
Differentiation grade					0.786
Tumor size					0.302
TNM					0.438
Intraoperative blood loss	−0.002	0.001	0.998	0.997–0.999	0.002
Combined organ resection	2.540	1.047	12.674	1.629–98.640	0.015

Adjusted OR estimated by the Cox model.

95% CI: 95% confidence interval, OR: odd ratio, and SE: standard error.

**Table 4:** Multivariate analysis of risk factors for blood transfusion in patients with POHb >100 g/L.

	Parameter estimate	SE	Adjusted OR	95% CI	*P* value
Age	0.799	0.322	2.224	1.184–4.178	0.013
Gender					0.481
Macroscopic type					0.829
Differentiation grade					0.113
Tumor size					0.957
Type of gastrectomy					0.402
TNM stage	−1.028	0.367	0.358	0.174–0.734	0.005
Longitudinal location					0.867
Intraoperative blood loss	−0.003	0.001	0.997	0.995–0.998	<0.001
Combined organ resection	1.105	0.518	3.019	1.094–8.333	0.033

Adjusted OR estimated by the Cox model.

95% CI: 95% confidence interval, OR: odd ratio, and SE: standard error.

**Table 5:** Univariate and multivariate Cox analysis for prognostic factors.

Risk factors	Categories	3-year overall survival rate (%)	Univariate analysis *P* value	Multivariate analysis		
				*P* value	OR	95% CI
Age (years)	≤60	64	0.001	0.005	1.308	1.084–1.579
	>60	56				

Gender	Male	59	0.191
	Female	63	

Type of gastrectomy	DG	66	<0.001	0.254
	TG	48		
	PG	54		

Combined organ resection	Yes	44	0.003	0.104
	No	61		

Longitudinal location	U	53	<0.001	0.934
	M	59		
	L	64		
	Whole	17		

Differentiation grade	Well/ moderate	70	0.009	0.078
	Poor/undifferentiated	58		

Macroscopic type	Types 0–2	68	<0.001	0.083
	Types 3–4	45		

Tumor size	≤4.5 cm	72	<0.001	<0.001	1.460	1.190–1.791
	>4.5 cm	48				

T stage	T1	87	<0.001
	T2	79	
	T3	65	
	T4	48	

N stage	N0	84	<0.001
	N1	68	
	N2	51	
	N3a	38	
	N3b	23	

TNM stage	I/II	83	<0.001	<0.001	2.607	2.087–3.256
	III	43				

Postoperative complications	Yes	52	0.110
	No	61	

Perioperative blood transfusion	Yes	49	<0.001	0.079
	No	64		

SD: standard deviation, DG: distal gastrectomy, TG: total gastrectomy, PG: proximal gastrectomy, U: upper, M: middle, and L: lower.
